# Ultrasound diagnosis of intrauterine device embedment complicated by bladder stone formation: a case report

**DOI:** 10.3389/fmed.2026.1872750

**Published:** 2026-08-17

**Authors:** Lina Kong, Kunming Pu, Xiao Tang, Xiachuan Qin

**Affiliations:** Department of Ultrasound, West China School of Medicine, Sichuan University Affiliated Chengdu Second People's Hospital, Chengdu Second People's Hospital, Sichuan University, Chengdu, Sichuan, China

**Keywords:** bladder stone, case report, intrauterine device embedment, ultrasound diagnosis, uterine perforation

## Abstract

**Objective:**

To investigate the diagnostic value of ultrasound in intrauterine device (IUD) embedment complicated by bladder stone formation and to discuss the clinical approach to diagnosis and management.

**Methods:**

We retrospectively analyzed the clinical data, ultrasound findings, imaging studies, and surgical course of a patient who underwent IUD removal due to family planning. Relevant literature was reviewed and discussed.

**Results:**

The patient presented for IUD removal and transvaginal color Doppler ultrasound revealed an abnormally positioned IUD with downward displacement and rotation, with one arm penetrating through the uterine serosa toward the bladder wall. Echogenic foci with acoustic shadowing were observed in the bladder. Pelvic CT confirmed IUD embedment with penetration through the uterus into the bladder and bladder stone formation. After infection control, the patient underwent multi-endoscope combined minimally invasive surgery. Intraoperative findings confirmed the ultrasound diagnosis. The patient recovered well postoperatively without complications.

**Conclusion:**

Transvaginal color Doppler ultrasound can clearly demonstrate the position and morphology of the IUD and its spatial relationship with the uterus and bladder. It is the preferred imaging modality for diagnosing IUD embedment complicated by bladder stone formation and provides important evidence for surgical planning.

## Introduction

Intrauterine device (IUD) insertion represents one of the most widely adopted methods of reversible contraception globally, with an estimated 150 million women currently using this modality, particularly in developing countries (1, 2). In China specifically, the number of women relying on IUDs for contraception exceeds the total number of users in the rest of the world combined, underscoring the profound public health significance of this contraceptive method in the region (3). The IUD is favored for its simplicity, safety, efficacy, cost-effectiveness, and reversibility, making it an ideal choice for long-term pregnancy prevention among women of reproductive age (1, 4).

Despite its generally favorable safety profile, IUD placement is not without complications. The spectrum of adverse events ranges from minor discomfort at the time of insertion to potentially life-threatening conditions (1). Common early complications include pelvic pain, abnormal uterine bleeding, vasovagal reactions, infection, menstrual irregularities, and expulsion (2). However, more serious complications such as uterine embedment and perforation, while rare, pose significant clinical challenges. The reported incidence of uterine perforation secondary to IUD migration or translocation ranges from 0.1 to 0.9%, though the true incidence likely remains underestimated because many affected patients are entirely asymptomatic, with discovery often occurring incidentally during investigations for unrelated pathologies (5). Large-scale active surveillance studies have documented uterine perforation rates of approximately 1.6 per 1,000 insertions, with risk varying according to practitioner experience, IUD type, and patient-specific anatomical factors (1, 6).

Uterine perforation represents the prerequisite for IUD migration, and once perforation occurs, the device may migrate to various extrauterine sites. Reported destinations for migrated IUDs include the abdominal cavity, omentum, broad ligament, rectosigmoid colon, peritoneum, appendix, adnexa, iliac vessels, subcutaneous tissue, and even the inguinal region (7, 8). Among all possible migration sites, the urinary bladder is recognized as the most common visceral organ involved, with an incidence of bladder wall penetration estimated between 0.05 and 1.3 per 1,000 women (3). Bjornerem et al. (5) demonstrated that approximately 2% of all IUD migrations involve the bladder. The anatomical proximity of the bladder to the anterior uterine wall, particularly in the setting of cesarean section scarring, renders this organ particularly vulnerable to IUD penetration.

The pathophysiology of spontaneous IUD migration remains incompletely understood, though several mechanisms have been proposed. Physiological uterine contractions, bowel peristalsis, and bladder contractions may all contribute to progressive displacement of a partially perforated device (9). The degree of uterine wall penetration at the time of insertion appears to be a critical determinant of subsequent migration risk (5). Secondary perforation, characterized by gradual pressure necrosis of the uterine wall due to chronic mechanical irritation, represents a delayed event that may manifest months to years after initial placement (1, 2). Risk factors for uterine perforation and subsequent migration are multifactorial and include postpartum insertion (particularly within 6 months), breastfeeding, multiparity, previous cesarean section, retroverted or malpositioned uterus, uterine fibroids, structural anomalies such as bicornuate or septate uteri, and insertion by inexperienced practitioners (2, 5, 8). Postpartum insertion substantially increases perforation risk due to reduced uterine consistency, diminished uterine wall dimensions, and intensified uterine contractility (5).

The clinical presentation of IUD migration into the bladder is highly variable. While some patients remain completely asymptomatic for prolonged periods, others experience a constellation of lower urinary tract symptoms including dysuria, frequency, urgency, hematuria, suprapubic pain, recurrent urinary tract infections, and abdominal pain (1, 5). A comprehensive literature review analyzing 115 cases of IUD migration to the urinary tract found that dysuria was the most common presenting symptom (33.0%), followed by hematuria (26.1%), pelvic pain (20.0%), urinary tract infection (15.6%), and various urinary tract symptoms (14.8%) (5). Notably, as many as 30% of patients with uterine perforation and IUD migration may be asymptomatic, contributing to delayed diagnosis and potential progression of complications (5).

Perhaps the most distinctive and clinically significant complication of intravesical IUD migration is secondary bladder stone formation. The IUD serves as a lithogenic foreign body, acting as a nidus for stone formation through chronic mucosal irritation, urine stasis, and potential infection (10). Approximately 70 cases of IUD migration to the bladder have been reported in scientific literature, with stone formation documented in roughly half of these cases (1, 11). A recent extensive literature review of 115 cases revealed that bladder stone formation occurred in 65.2% of patients with intravesical IUD migration, with the incidence increasing proportionally to the duration of migration (5). Established stone sizes have varied considerably, ranging from 1 to 8 cm in diameter (1). The interval between IUD insertion and clinical presentation is highly variable, spanning from as short as 6 months to several decades, with longer intervals correlating with increased stone formation risk (1, 12).

The diagnostic approach to suspected IUD migration with bladder involvement relies primarily on imaging modalities. Transvaginal ultrasound has emerged as the preferred initial imaging method, permitting excellent depiction of intravesical migrated IUDs and associated calculi (1, 11). In the comprehensive literature review by Sallami et al. (5) ultrasound was employed in 66.1% of cases, followed by plain radiography (61.7%), computed tomography (35.6%), and intravenous urography (11.3%). While ultrasound provides excellent visualization of the IUD position, morphology, and relationship to adjacent structures, its ability to definitively confirm complete transmural penetration into the bladder lumen may be limited, often necessitating complementary cross-sectional imaging with CT for comprehensive evaluation (5, 9). CT urography with three-dimensional reconstruction has been particularly advocated when urinary symptoms are present, as it may delineate potential erosion to the urinary system with greater precision (9).

Treatment of intravesical IUD migration with bladder stone formation is primarily surgical. The therapeutic modalities documented in the literature include cystoscopy (66.1%), laparoscopy (17.4%), cystolithotomy (9.5%), cystolitholapaxy (8.7%), open cystotomy (7.8%), and lithotripsy (13.8%, including laser lithotripsy in select cases) (5). The choice of surgical approach depends on the degree of bladder wall invasion, stone burden, IUD position, and associated complications such as fistula formation. Minimally invasive multi-endoscopic approaches combining hysteroscopy, laparoscopy, and cystoscopy have gained increasing favor, enabling simultaneous IUD extraction, stone fragmentation, and bladder wall repair with reduced surgical trauma (13). The prognosis is generally favorable, with 95.6% of reported cases achieving successful outcomes, though complications including vesicouterine and vesicoenteric fistulas have been documented in 4.3% of cases (5).

Despite the substantial body of literature addressing IUD migration and bladder stone formation, several clinical challenges persist. The rarity of this condition, combined with its often insidious and nonspecific presentation, contributes to frequent diagnostic delays and occasional misdiagnosis (3). Furthermore, the optimal timing of IUD placement following cesarean section remains a subject of ongoing debate, with current recommendations suggesting a minimum interval of 6 months to allow adequate uterine scar healing (8). Regular gynecological follow-up with ultrasound surveillance represents a critical yet frequently neglected component of post-insertion care, particularly in resource-limited settings where follow-up evaluations may be irregular or absent (1).

Herein, we report a case of IUD embedment with bladder stone formation occurring 14 years after cesarean section and subsequent IUD placement in a 39-year-old woman (Table 1). This case illustrates the critical role of transvaginal color Doppler ultrasound in establishing the preliminary diagnosis, the complementary value of pelvic CT in confirming transmural penetration, and the efficacy of multi-endoscope combined minimally invasive surgery in achieving complete resolution. Through detailed analysis of this case and comprehensive review of the relevant literature, we aim to enhance clinical awareness of this rare but serious complication and emphasize the importance of regular post-insertion surveillance, particularly among women with prior cesarean delivery.

**Table 1:** Chronological timeline of the patient's clinical course from cesarean section (2010) through 3-month postoperative follow-up (2026), including key clinical events, diagnostic evaluations, interventions, and outcomes.

Date/time period	Event	Clinical significance
2010	Lower segment cesarean section for cephalopelvic disproportion	Established risk factor for uterine scar formation and subsequent IUD complications
2011	V-shaped stainless-steel IUD placement (approximately 1 year post-cesarean)	Short interval between cesarean section and IUD placement; risk factor for embedment
2011–2025 (14 years)	No gynecological ultrasound follow-up; occasional mild urinary urgency and dysuria (2–3 episodes/year, self-resolving)	Absence of surveillance allowed progressive IUD migration; symptoms attributed to minor UTIs without medical evaluation
October 28, 2025	Last menstrual period	Proliferative phase at time of evaluation
Early November 2025	Presentation to community health center for routine IUD removal due to family planning change	Incidental discovery of IUD embedment during pre-removal evaluation
Early November 2025	Transvaginal color Doppler ultrasound (community health center): abnormal IUD position with right arm penetrating anterior uterine wall toward bladder; multiple echogenic foci with acoustic shadowing in bladder	Initial diagnostic imaging establishing preliminary diagnosis of IUD embedment with bladder involvement
Early November 2025	Referral to tertiary care center (Chengdu Second People's Hospital)	Escalation for definitive management
Early November 2025	Transvaginal color Doppler ultrasound (repeated at our institution): confirmed abnormal V-shaped IUD position at lower uterine segment; right arm penetrating uterine serosa toward bladder wall; multiple fixed bladder calculi (largest 1.8 cm); localized bladder wall thickening	Confirmatory imaging with detailed assessment of IUD-bladder relationship
Early November 2025	Pelvic CT with 1.25 mm thin-slice reconstruction: definitive demonstration of transmural IUD penetration through anterior uterine wall into bladder lumen; hyperdense calculus (1.8 × 1.2 cm) encasing intravesical IUD component; additional smaller calculi (5–8 mm); chronic inflammatory changes	Cross-sectional confirmation of transmural penetration; essential for surgical planning
Early November 2025	Laboratory evaluation: urinalysis positive for nitrites, occult blood, leukocytes; urine culture >105 CFU/mL *E. coli*; non-fasting glucose 7.29 mmol/L; normal coagulation, CBC, liver/renal function	Documented active urinary tract infection requiring preoperative treatment; metabolic screening
Preoperative days 1–5	Culture-directed antibiotic therapy (ceftriaxone 1 g IV q24h); resolution of infection confirmed by repeat urinalysis; fasting glucose 6.8 mmol/L, HbA1c 5.9% (diabetes excluded)	Preoperative optimization; infection control essential before surgical intervention
November 14, 2025	Combined multi-endoscopic surgery: hysteroscopic IUD removal, pelvic adhesiolysis, hysteroscopic endometrial lesion resection, bilateral tubal patency test, laparoscopic bladder repair, transurethral bladder lithotripsy and stone extraction, transurethral bladder lesion resection. Operative time: 187 min; EBL: 150 mL	Definitive surgical management; minimally invasive approach with complete IUD extraction, stone clearance, and bladder repair
Postoperative days 1–2	Pelvic drain removed (minimal serosanguinous output); continued IV antibiotics	Uncomplicated early postoperative course
Postoperative days 3–5	Transition to oral ciprofloxacin; Foley catheter maintained; discharge on postoperative day 5	Standard postoperative management; discharge criteria met
Postoperative day 7	Foley catheter removal after normal voiding trial	Successful bladder healing confirmed
1-month follow-up	Asymptomatic; normal urinalysis; transvaginal ultrasound: healing bladder wall, no residual stones or IUD fragments	Confirmation of successful surgical outcome
3-month follow-up	Continued asymptomatic status; normal urinary function; no recurrence	Long-term favorable outcome documented

## Case presentation

### Patient information

A 39-year-old multiparous woman of Han Chinese ethnicity was admitted to our hospital in November 2025 with a diagnosis of “IUD embedment discovered for half a day.” The patient was a full-time homemaker with a junior high school education level. She had resided in Chengdu, Sichuan Province for her entire life and had no history of occupational or environmental toxin exposure. The patient had regular menstrual cycles with a 28-day interval and 5-day duration, with her last menstrual period on October 28, 2025. She presented to a community health service center for routine IUD removal due to a change in family planning intentions. During the pre-removal evaluation, transvaginal color Doppler ultrasound revealed an abnormally positioned IUD with associated bladder abnormalities, prompting referral to our tertiary care center for further management and definitive treatment.

*Past medical history:* The patient underwent one lower segment cesarean section at an outside hospital in 2010 for cephalopelvic disproportion, followed by uncomplicated postoperative recovery. IUD placement was performed in 2011 at the same institution, approximately 1 year after cesarean delivery. The specific IUD type was documented as a V-shaped stainless-steel device, though the exact model and size were not available from the outside hospital records. Crucially, she had not undergone any gynecological ultrasound examination or IUD position assessment during the subsequent 14-year period, representing a significant deviation from recommended follow-up protocols (4, 14). She denied histories of hypertension, diabetes mellitus, thyroid disease, autoimmune disorders, bleeding diathesis, or previous urinary system diseases including nephrolithiasis or recurrent urinary tract infections. She had no history of pelvic inflammatory disease, sexually transmitted infections, or previous pelvic surgeries other than the cesarean section. Her obstetric history was otherwise unremarkable, with no history of spontaneous abortions, ectopic pregnancies, or infertility.

*Family history:* The patient reported no family history of gynecological malignancies, uterine fibroids, or pelvic inflammatory disease. Her mother had undergone uncomplicated hysterectomy for uterine fibroids at age 52. No family members had histories of urinary tract stones, chronic kidney disease, or congenital urogenital anomalies. There was no known family history of coagulation disorders or autoimmune conditions.

*Psychosocial history:* The patient was married for 18 years with a stable relationship. She had two children, both delivered by cesarean section (the second child in 2010). She was a non-smoker and denied alcohol consumption or recreational drug use. Her dietary habits were unremarkable, with no specific dietary restrictions or preferences. She reported adequate sleep (7–8 h nightly) and moderate stress levels related to family responsibilities. She had no regular exercise routine. The patient had basic medical insurance coverage through the urban resident basic medical insurance scheme, which provided partial reimbursement for medical expenses. She lived with her husband and children in a two-bedroom apartment with standard sanitation facilities. There was no history of domestic violence or significant psychosocial stressors. She reported strong social support from her extended family, who resided in the same district.

*Symptom review:* The patient reported that during the 14-year period since IUD placement, she had experienced occasional episodes of urinary urgency and mild dysuria, occurring approximately 2–3 times per year and typically resolving spontaneously within 1–2 days without medical intervention. She had attributed these symptoms to minor urinary tract infections or dietary factors and had not sought medical evaluation. She explicitly denied urinary frequency, gross hematuria, suprapubic or pelvic pain, abdominal distension, abnormal vaginal bleeding or discharge, dyspareunia, fever, or constitutional symptoms such as weight loss or fatigue. The absence of significant symptoms during the prolonged interval between IUD placement and diagnosis is consistent with the documented clinical behavior of this condition, wherein up to 30% of patients with uterine perforation and IUD migration may remain entirely asymptomatic, contributing to substantial diagnostic delays (1, 5).

### Physical examination

On admission, the patient appeared well-nourished and in no acute distress. Vital signs were stable: blood pressure 118/76 mmHg, heart rate 72 beats per min, respiratory rate 16 breaths per min, and body temperature 36.5 C. Her body mass index was calculated at 28.4 kg/m^2^, indicating obesity, which would subsequently impact the quality of abdominal palpation.

*Gynecological examination:* External genitalia were married-type with normal development and no visible lesions. The vagina was patent with normal rugated mucosa, no discharge, and no palpable masses. The cervix was of normal size, rosy in color, and smooth in appearance, without ectropion, polyps, or contact bleeding. Cervical motion tenderness and uterine tenderness were absent. The posterior fornix was not full and was non-tender, with no palpable nodularity. Due to abdominal adiposity and patient discomfort, bimanual palpation of the uterus and adnexa was technically limited and unsatisfactory for definitive assessment of size, position, and mobility. The uterus was vaguely palpable as anteverted and approximately normal in size, with no obvious adnexal masses detected.

*Systemic physical examination:* General examination revealed no pallor, jaundice, lymphadenopathy, or peripheral edema. Cardiovascular and respiratory examinations were unremarkable. Abdominal examination demonstrated moderate central obesity; the abdomen was soft and non-tender, with no palpable masses, organomegaly, or ascites. Costovertebral angle tenderness was absent bilaterally. No inguinal lymphadenopathy or hernias were identified.

### Auxiliary examinations

#### Laboratory tests

- *Urinalysis:* Positive nitrites (NIT), occult blood (BLD) (+), leukocytes (LEU) (3+), with negative glucose, protein, and ketones. These findings were consistent with active urinary tract infection, likely related to chronic bladder mucosal irritation and stone formation (1, 5). Urine culture was subsequently obtained and grew *Escherichia coli* >105 colony-forming units per milliliter, confirming the infectious etiology.
- *Blood glucose:* Non-fasting blood glucose 7.29 mmol/L (reference range 3.9–6.1 mmol/L), indicating impaired glucose tolerance or possible undiagnosed diabetes mellitus, which could potentially influence wound healing and infection risk. Fasting glucose and glycated hemoglobin were subsequently ordered for further metabolic assessment.
- *Coagulation profile:* Fibrinogen (Fbg) 3.62 g/L (reference range 2.0–4.0 g/L), within normal limits. Prothrombin time, activated partial thromboplastin time, and international normalized ratio were all within normal ranges, indicating no coagulopathy.
- *Hepatitis B serology:* HBsAb >1,000.000 mIU/mL (positive), HBcAb 210.000 IU/mL (positive), with negative HBsAg, HBeAg, and HBeAb. This pattern was consistent with resolved past hepatitis B infection with protective immunity, rather than active infection.
- *Blood type:* O type, RhD positive.
- *Complete blood count:* Hemoglobin 128 g/L, white blood cell count 7.2 × 10^9^/L with normal differential, platelet count 245 × 10^9^/L. No anemia, leukocytosis, or thrombocytopenia.
- *Comprehensive metabolic panel:* Liver function tests (alanine aminotransferase, aspartate aminotransferase, alkaline phosphatase, gamma-glutamyl transferase, total bilirubin, and albumin), renal function tests (creatinine, blood urea nitrogen, estimated glomerular filtration rate), and serum electrolytes (sodium, potassium, chloride, calcium, magnesium, and phosphorus) were all within normal reference ranges.
- *Vaginal discharge test:* Normal vaginal flora, negative for bacterial vaginosis, candidiasis, and trichomoniasis. pH 4.2, consistent with normal lactobacillary dominance.
- *Electrocardiogram:* Sinus rhythm at 68 beats per min, normal QRS axis, J-point elevation-type ST-segment elevation in leads V2–V4, consistent with benign early repolarization pattern. No ischemic changes, arrhythmias, or conduction abnormalities.

#### Imaging studies

##### Transvaginal color doppler ultrasound (performed at community health center and repeated at our institution)

The uterus was anteverted with an anteroposterior diameter of 4.0 cm, length of 6.8 cm, and width of 5.2 cm. Myometrial echogenicity was diffusely heterogeneous, suggestive of adenomyosis or prior surgical changes. A 1.0 cm × 0.7 cm well-circumscribed hypoechoic nodule with clear borders, regular morphology, and internal peripheral vascularity on color Doppler was identified in the anterior myometrial wall, consistent with a small leiomyoma. The endometrium was trilaminar with a double-layer thickness of 0.9 cm, appropriate for the proliferative phase of the menstrual cycle.

**Figure 1:**
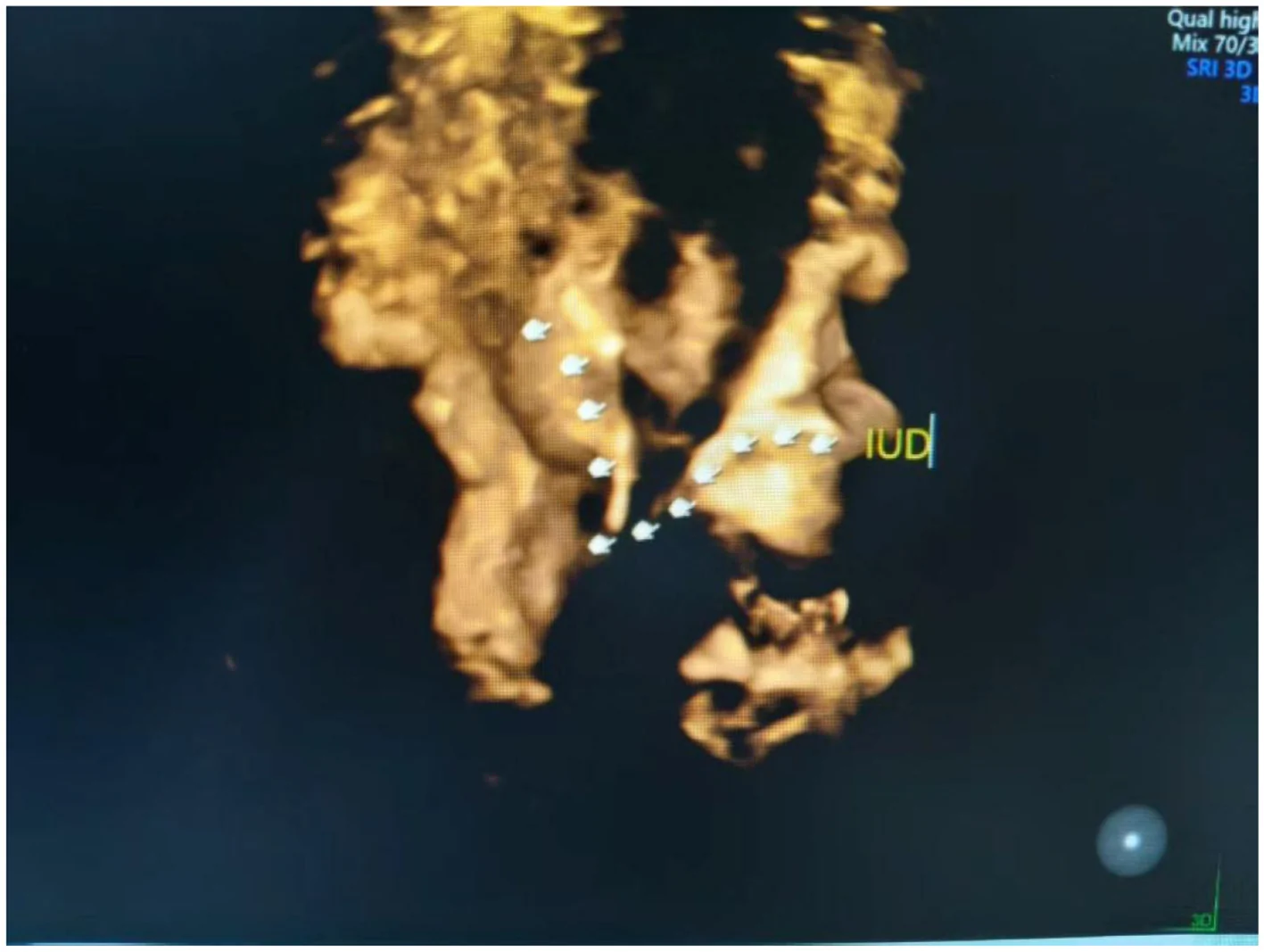
Transvaginal ultrasound image showing the abnormal position of the intrauterine device (IUD) at the lower uterine segment. Sagittal view demonstrating the V-shaped IUD with downward displacement and approximately 45-degree rotation from the expected coronal plane. The right arm extends anteriorly and superiorly, penetrating through the full thickness of the anterior uterine wall at the level of the cesarean section scar toward the bladder wall. The left arm terminates within the myometrium. Color Doppler image showing minimal vascularity along the tract of penetration consistent with a chronic inflammatory process rather than acute inflammation.

At the lower uterine segment, immediately superior to the internal cervical os, an intensely echogenic linear structure with posterior reverberation artifact was identified in an abnormal position, consistent with a V-shaped IUD. The device was rotated approximately 45 from the expected coronal plane. The left arm appeared to terminate within the myometrium, while the right arm extended anteriorly and superiorly, appearing to penetrate through the full thickness of the anterior uterine wall at the level of the presumed cesarean section scar (Figure 1). The arm tip was visualized extending beyond the uterine serosal surface toward the bladder wall, with the exact relationship to the bladder lumen indeterminate due to acoustic shadowing and possible inflammatory changes. Color Doppler demonstrated minimal vascularity along the tract of penetration, suggesting chronic rather than acute inflammatory process.

**Figure 2:**
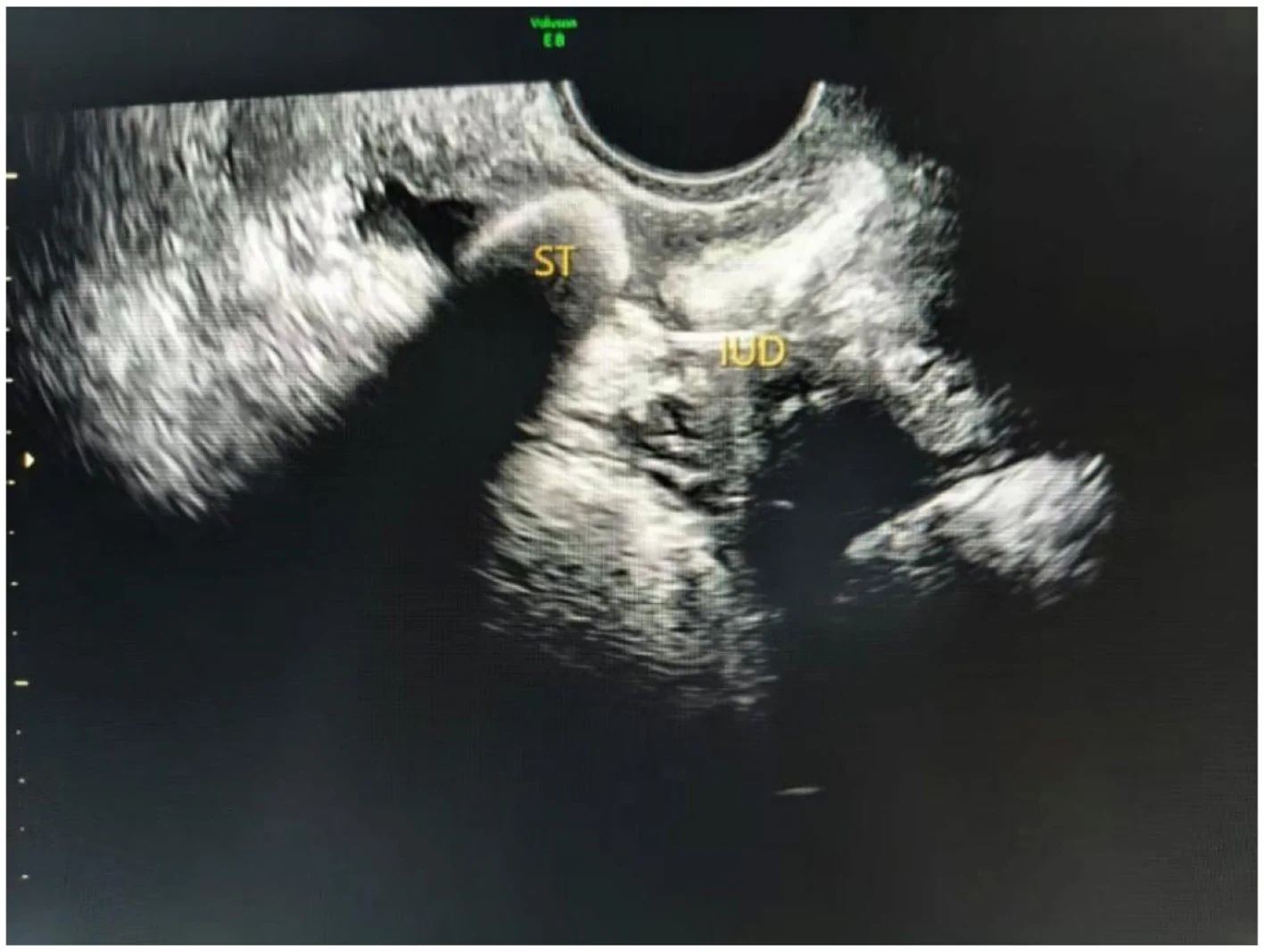
Transvaginal ultrasound image demonstrating bladder stone formation secondary to IUD migration. Sagittal view showing multiple intensely echogenic foci with prominent posterior acoustic shadowing (asterisks) within the bladder lumen. The stones are fixed in position at the site of IUD penetration and do not shift with changes in patient positioning or transducer pressure. Transverse view demonstrating localized bladder wall thickening with heterogeneous echogenicity at the stone attachment site, consistent with chronic inflammatory and fibrotic changes.

Corresponding to the site of IUD penetration, several intensely echogenic foci with prominent posterior acoustic shadowing were identified within the bladder lumen (Figures 1, 2). The largest stone measured approximately 1.8 cm in greatest dimension. The stones were fixed in position and did not shift with changes in patient positioning or transducer pressure, consistent with attachment to the bladder wall or encasement of the IUD. The bladder wall at this site appeared locally thickened (3.2 vs. 1.5 mm elsewhere) with heterogeneous echogenicity, suggesting chronic inflammatory and fibrotic changes (1, 12).

Bilateral adnexal regions were sonographically normal, with no masses, cysts, or free fluid. The rectouterine pouch was clear, with no evidence of hemoperitoneum or pelvic fluid collection.

*Ultrasound Impression* (1) Abnormal IUD position with embedment and probable transmural penetration toward the bladder; (2) Uterine solid nodule, most consistent with small leiomyoma; (3) Multiple bladder calculi, likely secondary to IUD migration and serving as a lithogenic nidus; (4) Localized bladder wall thickening with inflammatory changes.

##### Pelvic CT (plain scan with 1.25 mm thin-slice reconstruction)

CT imaging confirmed and extended the ultrasound findings. The V-shaped IUD was clearly visualized as a hyperdense metallic structure within the lower uterine segment. The right arm was definitively shown to penetrate through the full thickness of the anterior uterine wall, traverse the vesicouterine space, and enter the bladder lumen. The intravesical component of the IUD was partially encased by a hyperdense calculus measuring approximately 1.8 × 1.2 cm, with additional smaller calculi (5–8 mm) adjacent to the primary stone. The stones demonstrated typical calcific attenuation (850–1,100 Hounsfield units) with no evidence of uric acid composition. No gas was identified within the bladder wall or stones, arguing against emphysematous cystitis or fistulous communication with the gastrointestinal tract.

The bladder wall at the site of IUD penetration demonstrated focal thickening (4.5 mm) with mild surrounding fat stranding, consistent with chronic inflammatory changes. No active contrast extravasation was identified on the delayed phase images. The uterine serosa at the penetration site was irregular but without evidence of contained perforation or abscess formation. The previously identified myometrial nodule corresponded to a well-circumscribed, mildly enhancing lesion in the anterior uterine wall, consistent with a small leiomyoma.

No additional IUD fragments were identified within the peritoneal cavity or adjacent pelvic structures. No lymphadenopathy, ascites, or distant metastatic disease was present. The kidneys, ureters, and remainder of the urinary bladder were unremarkable.

*CT impression:* (1) IUD embedment with transmural penetration through the anterior uterine wall into the bladder lumen, confirmed; (2) Multiple bladder calculi with the largest measuring 1.8 cm, encasing the intravesical IUD component; (3) Localized chronic inflammatory changes of the bladder wall; (4) Small anterior uterine wall leiomyoma.

### Preoperative management

Following confirmation of the diagnosis, the patient was admitted for preoperative optimization. The active urinary tract infection was treated with culture-directed antibiotic therapy (ceftriaxone 1 g intravenously every 24 h) for 5 days, with resolution documented by repeat urinalysis showing negative nitrites, normal leukocyte esterase, and clearance of bacteriuria. Preoperative fasting glucose was 6.8 mmol/L and glycated hemoglobin was 5.9%, effectively excluding diabetes mellitus and explaining the mildly elevated non-fasting glucose as a stress-related or postprandial phenomenon. Anesthesiology consultation was obtained, and American Society of Anesthesiologists physical status classification was determined as ASA II due to obesity and the resolved urinary tract infection.

Informed consent was obtained after detailed discussion of the diagnosis, proposed multi-endoscopic surgical approach, potential risks including bleeding, infection, bladder or uterine injury requiring open conversion, urinary retention, and the possibility of incomplete stone clearance requiring secondary procedures. The patient expressed understanding and provided written consent. Preoperative bowel preparation with clear liquid diet and sodium phosphate enema was administered the evening before surgery.

### Treatment and surgical course

On November 14, 2025, the patient was taken to the operating room and underwent the following combined procedures under general anesthesia with endotracheal intubation: hysteroscopic IUD removal, pelvic adhesiolysis, hysteroscopic endometrial lesion resection, bilateral tubal patency test, laparoscopic bladder repair, transurethral bladder lithotripsy and stone extraction, and transurethral bladder lesion resection. Total operative time was 187 min. Estimated blood loss was 150 mL. No intraoperative complications occurred, and no blood transfusion was required.

#### Intraoperative findings

**Figure 3:**
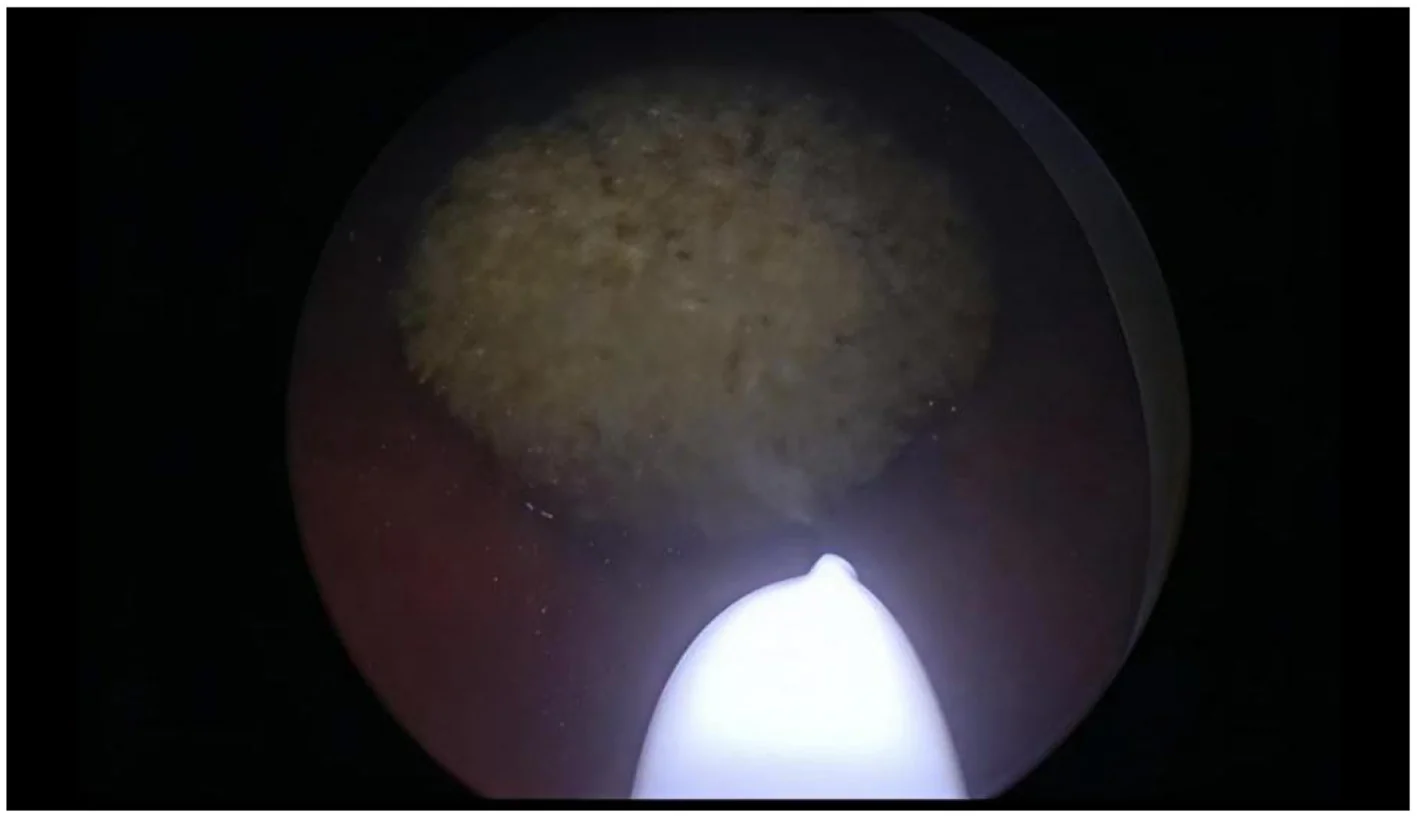
Intraoperative cystoscopic image, showing the presence of bladder stones.

**Figure 4:**
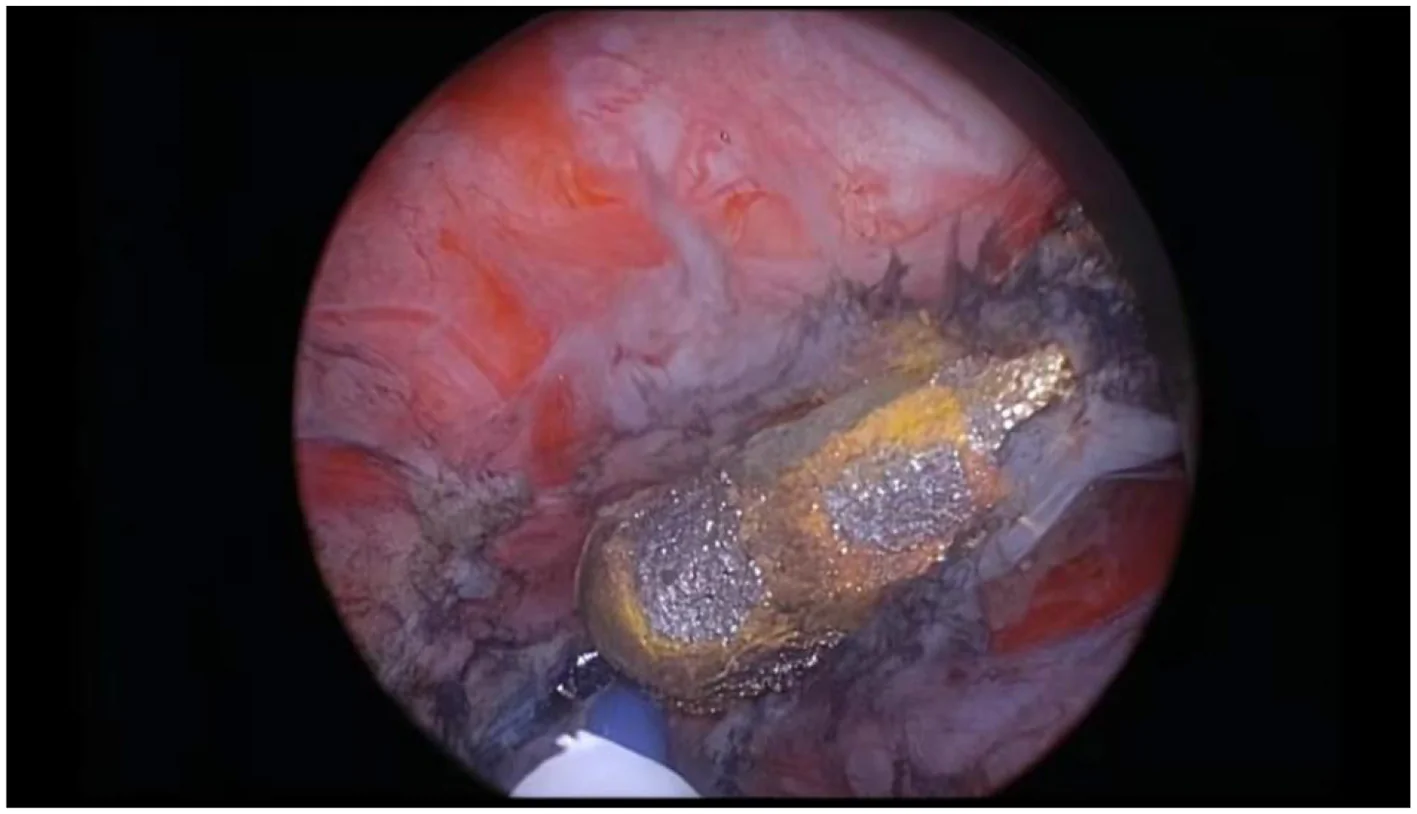
Intraoperative cystoscopic image after holmium laser lithotripsy, showing the remnant of an intrauterine device.

1. *Laparoscopic exploration:* Upon pneumoperitoneum establishment and trocar placement, the abdominal cavity was systematically inspected. Portions of small bowel and associated mesentery were found densely adherent to the left pelvic wall and peritoneum, likely secondary to the prior cesarean section. These adhesions were carefully lysed using blunt and sharp dissection with ultrasonic shears, restoring normal anatomical relationships. The lower uterine segment was densely adherent to the superior bladder surface over an area approximately 3 × 2 cm, with obliteration of the normal vesicouterine space. Gentle dissection with hydrodissection and blunt technique gradually separated these structures, revealing the site of IUD penetration. The uterus was of normal size with unremarkable external serosal surface except for the focal defect at the anterior lower segment. The bilateral fallopian tubes and ovaries appeared normal in size, shape, and position, with no masses, cysts, or inflammatory changes.
2. *Transurethral cystoscopy (F22 rigid cystoscope):* Under direct vision, the urethra was patent without strictures, stones, or neoplasms. The bladder neck posterior lip was mildly elevated, a common finding in multiparous women. Systematic inspection of the bladder mucosa revealed normal trigone and ureteric orifices bilaterally, with clear efflux of urine. On the anterior bladder wall, approximately 2 cm superior to the bladder neck and slightly to the right of midline, an ovoid, yellow-brown calculus was identified (Figure 3). The stone was hard in consistency, with a maximum diameter of 1.0 cm (smaller than the 1.8 cm preoperative measurement, likely due to CT overestimation or partial fragmentation during patient positioning and manipulation). The stone partially encased a metallic wire-like structure, which was identified as the intravesical component of the V-shaped IUD (Figure 4). The stone and encased IUD were surrounded by flocculent whitish-yellow material and areas of necrotic-appearing mucosa, consistent with chronic inflammatory debris and granulation tissue. The surrounding bladder mucosa was hyperemic and edematous but without evidence of active purulent exudate or neoplastic change. No additional stones or mucosal abnormalities were identified.
3. *Hysteroscopic examination (26F continuous-flow operative hysteroscope):* After cervical dilation to 9 mm, the hysteroscope was introduced into the uterine cavity. The endometrium appeared normal in thickness and color. At the lower uterine segment, immediately above the internal os, a V-shaped metallic IUD was identified. The left arm was partially embedded in the myometrium but with visible endometrial surface contact. The right arm was deeply embedded in the anterior uterine wall myometrium, with only a small segment visible at the cavity surface. The embedding site corresponded precisely to the anterior lower uterine segment, consistent with the cesarean section scar site. Using hysteroscopic scissors and grasping forceps, the visible portions of the IUD were carefully grasped and gentle traction was applied. With combined laparoscopic visualization of the external uterine surface to prevent perforation, the embedded right arm was gradually extracted from the myometrium. The IUD was removed in one piece without fragmentation. The endometrial cavity was then systematically inspected, and a small, raised, hyperemic lesion measuring 0.6 cm was identified in the right cornual region. This lesion was resected using hysteroscopic electrosurgical loop and sent for histopathological examination. Bilateral tubal ostia were cannulated with a 3F catheter, and methylene blue dye was injected bilaterally, demonstrating free spill from both tubes, confirming patency.
4. *Laparoscopic bladder repair:* Following IUD removal and hysteroscopic completion, attention was returned to the laparoscopic field. The bladder wall defect created by IUD penetration was identified as a 1.5 cm longitudinal laceration in the detrusor muscle with intact mucosa. The defect was repaired in two layers using 3-0 absorbable suture (Vicryl) in a running fashion, with the first layer approximating the detrusor muscle and the second layer imbricating the seromuscular layer. The repair was tested for watertight integrity by retrograde bladder filling with 300 mL of sterile saline *via* the transurethral catheter, with no leakage observed. A closed suction drain was placed in the pelvis.
5. *Transurethral stone management:* Following bladder repair, the transurethral resectoscope was reintroduced. The stone encasing the IUD had already been largely disrupted during the extraction process. Residual stone fragments were fragmented using pneumatic lithotripsy and extracted using Ellik evacuator. The bladder mucosa at the stone site was resected to remove the inflammatory granulation tissue, and the base was cauterized to achieve hemostasis. Final inspection confirmed complete stone clearance, intact bladder repair, and no active bleeding.

The surgery was completed successfully. The patient was extubated in the operating room and transferred to the post-anesthesia care unit in stable condition. The pelvic drain was removed on postoperative day 2 after minimal serosanguinous output. The transurethral Foley catheter was maintained for 7 days to allow bladder healing, then removed after a normal voiding trial. Postoperative antibiotics (ceftriaxone) were continued for 48 h and transitioned to oral ciprofloxacin for 5 days. The patient was discharged home on postoperative day 5 with instructions for follow-up.

*Histopathological findings:* The resected endometrial lesion demonstrated benign endometrial polyp with focal simple hyperplasia without atypia. The inflammatory debris from the bladder stone site showed necrotic tissue with chronic inflammatory cell infiltrate, fibrosis, and calcification, consistent with foreign body reaction to the IUD.

*Postoperative course and follow-up:* The patient recovered uneventfully. At the 1-month follow-up visit, she reported complete resolution of urinary symptoms, with no dysuria, urgency, or frequency. Repeat transvaginal ultrasound demonstrated a normally healing bladder wall with no residual stones or IUD fragments. The uterus was normal in size with a small residual anterior wall leiomyoma unchanged from preoperative imaging. Urinalysis was normal. At the 3-month follow-up, she remained asymptomatic with normal urinary function and no evidence of recurrence.

## Discussion

### Etiological analysis

IUD embedment with ectopic migration into the bladder and subsequent stone formation is a rare but serious complication of IUD placement. Its occurrence results from multiple contributing factors that interact over prolonged periods. Based on this case and comprehensive domestic and international literature (1–3, 5–10, 12–16), the main risk factors include the following three aspects:

1. *Abnormal uterine anatomy after cesarean section:* The patient underwent cesarean section in 2010 and IUD placement in 2011, representing a relatively short interval of only 1 year between uterine surgery and contraceptive device insertion. This temporal proximity is particularly significant because cesarean section scars undergo a prolonged healing process, with complete myometrial remodeling and restoration of tensile strength requiring a minimum of 6 months and often extending considerably longer (4, 8). Poor healing and thinning of the myometrium at the anterior uterine wall incision site provided an important anatomical basis for IUD embedment and subsequent penetration. The cesarean scar site is characterized by diminished blood supply, altered collagen deposition, and weak tissue repair capacity, creating a zone of biomechanical vulnerability (4). Long-term mechanical irritation from the IUD, particularly from a V-shaped device with sharp arm ends, can progressively erode the attenuated myometrium at the scar site, eventually penetrating through the serosa and migrating toward the adjacent bladder. This mechanism aligns with the concept of secondary perforation, wherein chronic pressure necrosis gradually erodes the uterine wall over months to years rather than occurring at the time of insertion (1, 2). The anatomical proximity of the bladder to the anterior lower uterine segment, where cesarean incisions are typically performed, renders this organ particularly susceptible to IUD penetration in post-cesarean patients (3). Multiple studies have documented that cesarean section history constitutes a significant risk factor for uterine perforation and subsequent IUD migration, with the incidence of migration to the bladder specifically estimated between 0.05 and 1.3 per 1,000 women (3, 5).
2. *Long-term IUD placement without regular follow-up*: The patient had not undergone gynecological ultrasound examination for 14 years after IUD placement, representing a critical failure in post-insertion surveillance. This prolonged absence of follow-up precluded early detection of IUD displacement, embedment, or migration, allowing the condition to progress from an initially manageable stage to a complex complication requiring multi-modal surgical intervention. As a foreign body, the long-term embedment of the IUD in the myometrium with continuous mechanical irritation of the bladder wall caused chronic mucosal injury, inflammatory changes, and ultimately lithogenic conditions leading to stone formation (1, 5, 10–12, 14, 15). The relationship between duration of migration and stone formation risk has been well established in the literature, with longer intervals correlating with substantially increased stone formation rates (1, 5). In a comprehensive review of 115 cases of IUD migration to the urinary tract, bladder stone formation occurred in 65.2% of patients, with the incidence increasing proportionally to the duration of migration (5). The 14-year interval in this case falls within the documented range of 6 months to several decades reported in the literature (1, 12). The IUD serves as a lithogenic nidus, promoting stone formation through multiple mechanisms including chronic mucosal irritation, urine stasis around the foreign body, potential infection with urease-producing organisms, and provision of a physical scaffold for crystal nucleation and aggregation (10, 11). The stone that encased the IUD in this case measured up to 1.8 cm on preoperative imaging and was composed of hard, calcified material with surrounding inflammatory debris, consistent with the chronic lithogenic process described in the literature (1, 11).
3. *Physiological uterine contractions and device characteristics:* The uterine body exhibits cyclical contractile activity throughout the menstrual cycle, with particularly intense contractions during menstruation and in the immediate postpartum period (5). These physiological contractions exert continuous mechanical pushing forces on the IUD, causing gradual downward displacement and rotation over time. In a normally positioned IUD, these forces are accommodated within the endometrial cavity; however, when the device is positioned near a zone of myometrial weakness, such as a cesarean scar, the contractions can drive the IUD progressively into the myometrium and eventually through the serosa (2). In this case, the IUD was V-shaped with sharp arm ends, a design feature that more easily pierced the attenuated myometrium at the cesarean scar site and increased the risk of ectopic migration. The V-shaped configuration concentrates mechanical stress at the arm tips, facilitating tissue penetration when resistance is diminished, as in scarred or thin myometrium (12, 15). Additionally, the patient's uterine leiomyoma, identified on imaging as a 1.0 × 0.7 cm hypoechoic nodule in the anterior wall, may have contributed to altered uterine contractility patterns and cavity distortion, potentially influencing IUD positioning and migration dynamics, though this association remains speculative and warrants further investigation (2).

### Clinical value of ultrasound diagnosis

Transvaginal color Doppler ultrasound has established itself as the preferred initial imaging modality for diagnosing IUD-related complications, with advantages of being non-invasive, real-time, repeatable, cost-effective, and high-resolution (4). It can clearly demonstrate the fine structures of the uterine myometrium, endometrial cavity, IUD morphology and position, and adjacent pelvic organs including the bladder (1, 11). In the comprehensive literature review by Sallami et al. (5), ultrasound was employed in 66.1% of cases of IUD migration to the urinary tract, reflecting its central role in the diagnostic algorithm. In this case, transvaginal ultrasound combined with three-dimensional reconstruction clearly demonstrated the abnormal position of the V-shaped IUD at the lower uterine segment, confirmed that its right arm had penetrated through the uterine serosa toward the bladder wall, and simultaneously identified the typical sonographic appearance of multiple echogenic foci with acoustic shadowing in the bladder, providing the core basis for preliminary clinical diagnosis.

The sonographic features of IUD embedment with bladder stone formation are characteristic and, when recognized, highly suggestive of the diagnosis (1, 12). These features include: (1) The echogenic IUD is displaced from its normal position within the uterine cavity, partially or completely embedded in the myometrium, or even penetrating through the serosa with close apposition to or invasion of the bladder wall; (2) Echogenic foci with posterior acoustic shadowing are seen in the bladder at sites adjacent to or surrounding the IUD. The stones typically do not change position with body movement because they encase the IUD and are adherent to the bladder wall, a feature that distinguishes them from free-floating bladder calculi (5, 12); and (3) In some patients, localized bladder wall thickening with heterogeneous echogenicity may be seen, suggesting chronic mucosal injury, inflammatory infiltration, and granulation tissue formation (1, 12). The color Doppler component may demonstrate increased vascularity in the bladder wall surrounding the stone, reflecting active inflammation (4).

Despite its considerable diagnostic utility, ultrasound has well-recognized limitations in determining whether the IUD has completely penetrated the bladder wall and entered the bladder lumen (5, 9). In this case, ultrasound could not definitively establish the full-thickness penetration relationship between the IUD and bladder wall, necessitating pelvic CT for further confirmation. This limitation arises from the inherent difficulty in distinguishing between close apposition of the IUD to the bladder wall vs. actual transmural penetration and intravesical extension, particularly when inflammatory changes and adhesions obscure tissue planes (5). The combination of ultrasound and CT improves diagnostic accuracy and provides more comprehensive imaging information for surgical planning. CT urography with three-dimensional reconstruction has been particularly advocated when urinary symptoms are present, as it may delineate potential erosion to the urinary system, identify associated complications such as fistulas, and provide superior anatomical detail for preoperative planning (9). In this case, pelvic CT confirmed the ultrasound findings, definitively established transmural penetration into the bladder, and identified the associated bladder stone, thereby guiding the multi-endoscopic surgical approach.

The differential diagnosis of IUD migration with bladder involvement includes primary bladder stones, bladder tumors with calcification, foreign bodies from other sources, and chronic cystitis with calcification (16). The clinical history of IUD placement, combined with the characteristic imaging findings of an echogenic linear structure contiguous with the uterus and extending into the bladder, typically permits accurate diagnosis. However, in cases where the IUD has been forgotten or the patient is unaware of its presence, the diagnosis may be considerably delayed, with the condition occasionally discovered only during surgery for presumed primary bladder pathology (3).

### Clinical management strategy

The treatment of IUD embedment with bladder stone formation is primarily surgical, with the therapeutic approach tailored to the degree of bladder wall invasion, stone burden, IUD position, presence of adhesions, and associated complications (5, 10, 15, 16). Preoperative active control of urinary tract infection is essential to prevent infection spread, reduce perioperative complications, and optimize surgical outcomes. In this case, the patient presented with active urinary tract infection evidenced by positive nitrites, occult blood, and leukocytes on urinalysis, necessitating antibiotic therapy before surgical intervention.

The core surgical principles include complete IUD extraction, clearance of bladder stones, repair of damaged uterine and bladder walls, restoration of normal anatomy, and minimization of surgical trauma (10, 15, 16). Various surgical modalities have been documented in the literature, including cystoscopy (66.1%), laparoscopy (17.4%), cystolithotomy (9.5%), cystolitholapaxy (8.7%), open cystotomy (7.8%), and lithotripsy (13.8%, including laser lithotripsy in select cases) (5). The choice of approach depends on the complexity of the case, surgeon expertise, and available resources.

This case employed multi-endoscope combined minimally invasive surgery, integrating laparoscopy, hysteroscopy, and cystoscopy in a single operative session. This approach offered several distinct advantages: (1) Laparoscopy permitted visualization and lysis of dense pelvic adhesions between the bowel, left pelvic wall, and lower uterine segment, safe dissection of the bladder from the uterus, and repair of the bladder wall defect after stone and IUD removal; (2) Hysteroscopy enabled direct visualization of the IUD within the uterine cavity and myometrium, facilitating safe extraction of the embedded portion and inspection of the endometrial cavity; and (3) Cystoscopy allowed transurethral access to the bladder for stone fragmentation, removal of the intravesical IUD component, and inspection of the bladder mucosa for additional lesions. The intraoperative findings confirmed the preoperative imaging diagnosis: an ovoid stone encasing the IUD on the anterior bladder wall, hard in consistency with a maximum diameter of 1.0 cm, surrounded by flocculent material and necrotic tissue indicative of chronic inflammatory changes.

The multi-endoscopic approach exemplifies the modern paradigm of minimally invasive surgery for this condition, reducing surgical trauma, minimizing postoperative pain, accelerating recovery, and avoiding the morbidity associated with open surgery (13). Shin et al. (13) demonstrated that laparoscopic removal represents a minimally invasive alternative to open surgery for IUD embedded in the bladder wall with stone formation, with excellent outcomes and rapid recovery. The successful application of this approach in our case, with complete resolution and absence of postoperative complications, supports its broader adoption for appropriately selected patients.

Furthermore, clinical attention should be paid to health management after IUD placement as a critical preventive strategy. Women with IUDs should be informed about the importance of regular gynecological ultrasound follow-up, with examinations recommended at 1, 3, and 6 months, and annually thereafter, or more frequently if symptoms develop (4, 14). Women with a history of cesarean section are advised to consider IUD placement only after a minimum interval of 6 months postoperatively, allowing sufficient time for uterine scar healing and restoration of myometrial integrity (8). If symptoms such as urgency, dysuria, frequency, hematuria, abdominal pain, or abnormal vaginal bleeding occur, prompt medical attention is recommended for early detection and management of IUD-related complications (14). The substantial delay in diagnosis in this case, attributable to 14 years without follow-up, underscores the critical importance of patient education and adherence to surveillance protocols.

The prognosis following surgical management of IUD migration with bladder involvement is generally favorable. In the comprehensive literature review by Sallami et al. (5), successful outcomes were achieved in 95.6% of cases, with complications including vesicouterine and vesicoenteric fistulas documented in 4.3% of cases. Long-term follow-up is recommended to monitor for recurrence of stones, bladder wall healing, and restoration of normal urinary function. In this case, the patient recovered well postoperatively without complications, consistent with the favorable outcomes reported in the literature.

## Limitations

This case report has several inherent limitations that should be acknowledged. First, as a single-case report, the findings have restricted generalizability and cannot be extrapolated to the broader population of women with IUDs or those with IUD migration. The specific combination of risk factors in this patient (cesarean section history, short interval to IUD placement, V-shaped device, 14-year absence of follow-up) represents a unique constellation that may not be representative of typical clinical presentations. Second, certain clinical data were unavailable due to the prolonged interval since the initial IUD placement. The exact model and size of the V-shaped IUD could not be retrieved from the outside hospital records, limiting precise characterization of the device. Third, the mechanism of initial IUD placement—whether the device was already partially embedded at insertion or migrated secondarily over time—cannot be definitively established retrospectively. Fourth, the patient reported only occasional mild urinary symptoms over 14 years, and the exact timeline of symptom progression was subject to recall bias. Fifth, histopathological examination of the bladder wall at the penetration site was not performed, as the inflammatory debris was managed through transurethral resection rather than open biopsy, limiting detailed pathological characterization of the chronic inflammatory process. Sixth, the absence of a formal patient-reported outcome measure (PROM) limits the objective assessment of quality-of-life improvement following surgery. Finally, the 3-month follow-up period, while demonstrating favorable short-term outcomes, is insufficient to assess very long-term complications such as bladder wall weakening, uterine scar dehiscence, or stone recurrence; extended follow-up would strengthen the evidence for sustained surgical success.

## Conclusion

This case report demonstrates that transvaginal color Doppler ultrasound is the preferred initial imaging modality for diagnosing IUD embedment with bladder stone formation, providing critical information for clinical diagnosis and surgical planning, while pelvic CT is essential for confirming transmural penetration and guiding operative strategy. The multi-endoscope combined minimally invasive approach proved highly effective for this rare complication. Most importantly, this case underscores the necessity of regular gynecological follow-up after IUD placement, particularly in women with prior cesarean section, to enable early detection and prevent progression to complex surgical disease.

## Data Availability

The original contributions presented in the study are included in the article/supplementary material, further inquiries can be directed to the corresponding author.
